# Microbial responses to transient shock loads of quaternary ammonium compounds with different length of alkyl chain in a membrane bioreactor

**DOI:** 10.1186/s13568-018-0649-5

**Published:** 2018-07-17

**Authors:** Xingran Zhang, Jinxing Ma, Mei Chen, Zhichao Wu, Zhiwei Wang

**Affiliations:** 1 State Key Laboratory of Pollution Control and Resource Reuse, Shanghai Institute of Pollution Control and Ecological Security, School of Environmental Science and Engineering, Tongji University, 1239 Siping Road, Shanghai, 200092 China; 2 UNSW Water Research Centre, School of Civil and Environmental Engineering, University of New South Wales, Sydney, NSW 2052 Australia

**Keywords:** Quaternary ammonium compounds, Reactive oxygen species, Microbial viability, Membrane bioreactor, Wastewater treatment

## Abstract

Extensive applications of quaternary ammonium compounds (QACs) in household and industrial products inevitably lead to their release into wastewaters; however, little attention has been paid to the acute effects on activated sludge. In this work, we investigated the responses of microorganisms in a membrane bioreactor (MBR) to transient shock loads of QACs with different alkyl chain length and their impacts on MBR performance. Results showed that QACs affected microbial viability and caused damage to key enzymes (e.g., ammonium monooxygenase and nitrite oxidoreductase), inhibiting organic matter degradation and nitrogen removal. The presence of QACs also caused negative influences on dehydrogenase activity, catalase and superoxide dismutase, thus increasing the production of reactive oxygen species. Moreover, QACs with longer alkyl chains and/or benzyl groups bonded to the nitrogen atom could induce a more severe damage to cell integrity and microbial viability. The interaction with QACs also induced the release of organic matters and the changes of adhesion properties of microbial products, resulting in aggravated membrane fouling in MBRs. Our results demonstrate the acute negative effects of QACs on activated sludge, and special attention should be paid to the performance of biological wastewater treatment processes subject to the shock loads of QAC-bearing industrial streams.

**Electronic supplementary material:**

The online version of this article (10.1186/s13568-018-0649-5) contains supplementary material, which is available to authorized users.

## Introduction

The growing concerns over the scarcity of freshwater sources and increasingly stringent environmental standards have incented advanced treatment and reclamation of domestic wastewater. Of the technologies available, membrane bioreactor (MBR) has gradually gained popularity as a result of its superior solid–liquid separation performance that provides high-quality effluent suitable for the downstream polishing (e.g., deionization) (Judd 2008; Monsalvo et al. 2014; Wang et al. 2014). Driven by the advantages such as low footprint, high nutrient removal efficiency and, if designed appropriately, ease of operation and maintenance, the annual growth rate of MBRs in the global market is assessed to be ca. 15% with the cumulative treatment capacity of large-scale MBR plants around the world (i.e., the capacity of each plant > 100,000 m^3^/day) reaching 5 million m^3^/day by 2017 (Judd 2016; Meng et al. 2017).

While the great success in commercialization corroborates that the MBR technology has become more and more sophisticated in domestic wastewater treatment with the intensive R&D efforts (Kim et al. 2011; Ma et al. 2015; McCarty et al. 2011), there is, surprisingly, limited consideration given to the increasing proportion of the industrial streams in domestic wastewater and its impacts on the performance of MBRs especially during the urbanization of developing countries such as China (Hubacek et al. 2009). It is initially thought that MBRs should have versatility in resisting to the shock loads due to the high biomass concentration and retention capacity of the membrane. However, recent studies indicate that even salt stress could lead to the deterioration of MBR performance, which takes days for the system to regain its stability (Yogalakshmi and Joseph 2010). As such, the periodical shock loads of the chemical industry wastes are also expected to cause adverse effects on the activity of microorganisms in MBRs. A comprehensive investigation on the microbial responses to the chemical shock loads and the consequent impacts on the performance of MBRs is therefore of great importance for the optimization of the system operation and development of controlling strategies.

Quaternary ammonium compounds (QACs) are a group of cationic surfactants widely used in fabric softening, disinfection, preservation and destaticization (Lara-Martín et al. 2010; Ruan et al. 2014), and their toxicity to a variety of model organisms has been documented in numerous studies (e.g., EC_50_ of *Chlorella vulgais*: 0.203 mg/L; EC_50_ of zebrafish liver cells: 850 μg/L and EC_50_ of *Aliivibrio fischeri*: 1.0 mg/L) (Christen et al. 2017; Di Nica et al. 2017; Zhu et al. 2010). It is estimated that about 75% of the QACs consumed annually are released into wastewater treatment systems (Ismail et al. 2010). The rinse water from hospitals, laundries and poultry processing facilities, and roof runoff are likely the point sources (Ruan et al. 2014; Tezel et al. 2007), causing transient shock loads of QACs in wastewater treatment plants. Although extensive information on the occurrence and fate of QACs in the aquatic environment has indicated that biomass sorption via ion exchange, ion pairing and/or hydrophobic bonding largely accounts for the removal of QACs in wastewater treatment plants (a low-speed degradation resulting in the accumulation of QACs in waste activated sludge) (Li et al. 2014; Tezel and Pavlostathis 2015; Zhang et al. 2015), there is limited knowledge on the subsequent microbial behaviors on exposure to different kinds of QACs (e.g., monoalkonium and benzalkonium compounds). Recent evidence indicates that the immobilized QACs on uniform planes (e.g., membranes) could retard microbial growth through contact-killing (Chen et al. 2017; Zhang et al. 2016). However, there is no detailed study on the change of cell viability and microbial metabolisms due to the acute response to QAC shock loads in MBRs treating domestic wastewater.

The aim of this study is therefore to investigate the acute responses of microorganisms in an MBR undergoing short-term elevated concentrations of QACs. Key questions to be addressed in the present work include (i) How do the transient shock loads of QACs affect the biodegradation and nitrogen metabolism? (ii) What is the relationship between microbial intracellular responses and QACs with different length of alkyl chain? (iii) What is the prevailing mechanism involved in the release of microbial products that leads to membrane fouling in the presence of QACs?

## Materials and methods

### Reagents

Unless otherwise stated, all chemicals and reagents used in this study were of analytical grade and used as received without further purification. Sodium acetate (NaAc), ammonium chloride (NH_4_Cl) and sodium nitrate (NaNO_3_) were received from Aladdin (China). Four kinds of QACs, three monoalkonium and one benzalkonium compounds, were purchased from Sigma Aldrich. Chemical structures and other relevant information of the four kinds of QACs are listed in Table 1. Stock solutions (1.5 mM) of each individual QAC were prepared in deionized (DI) water.

**Table 1:** Information of the four kinds of QACs used in this study

QACs	Abbreviations	Molecular structure	Chemical formula	Molecular weight (g/mol)	Purity (%)
Octyl trimethyl ammonium chloride	ATAC C8		C_11_H_26_NCl	207.5	≥ 97.0
Dodecyl trimethyl ammonium chloride	ATAC C12		C_15_H_34_NCl	263.5	≥ 98.0
Hexadecyl trimethyl ammonium chloride	ATAC C16		C_20_H_44_NCl	333.5	≥ 98.0
Hexadecyl benzyl dimethyl ammonium chloride	BAC C16		C_25_H_46_NCl	395.5	≥ 98.0

### Sludge samples and QAC exposure

The experimental procedures are shown in Additional file 1: Fig. S1. Sludge samples (100 mL, the concentration of suspended solid (SS) was predetermined before test) were collected from a pilot-scale anoxic/oxic MBR system treating domestic wastewater (see detailed information about this MBR in Additional file 1: Section S1) followed by centrifugation (3500×*g*, 5 min) to remove the supernatant. The sediments were washed twice with 10 mM phosphate buffer saline (PBS, pH 7.4) (Additional file 1: Fig. S1). Subsequently, the treated samples were resuspended with nutrient solutions in five flasks (one as control and the other four for QAC tests) with the biomass concentrations of 1.0 g-SS/L. The total volume of sample was controlled at 100 mL and the compositions of nutrient solutions were as follows: CH_3_COONa 320 mg/L, NH_4_Cl 77 mg/L. An elevated concentration (i.e., 15 μmol/g-SS, higher than the background value of 0.1–1 μmol/g-SS) was chosen for each QAC according to the previous studies (Lara-Martín et al. 2010; Li et al. 2014; Li and Brownawell 2010), with an exposure duration of 2 h to simulate the shock loads of QACs. Magnetic stirring (300 rpm) was applied to maintain the DO concentration at 2–3 mg/L. After that, samples were subject to centrifugation (3500×*g*, 5 min) with the remaining pellets washed twice and re-suspended in 10 mM PBS for the following measurements unless otherwise stated (Additional file 1: Fig. S1).

### Determination of biodegradation and nitrogen metabolisms

To investigate the change in the biodegradation behaviors of organic matters, 400 mg/L of NaAc was added into the mixtures following 2 h of QAC exposure (Additional file 1: Fig. S1). Dissolved oxygen (DO) concentrations were maintained at ~ 5.0 mg/L. Mixed liquor samples withdrawn at predetermined intervals (0, 15, 30, 60 and 120 min) were immediately filtered with 0.45-μm PTFE membranes prior to the measurement of NaAc concentrations by gas chromatography (6890 N, Agilent, US) with a flame ionization detector (Mei et al. 2014). The biodegradation rates were calculated via the derivation of the concentration profiles in the initial 30 min to minimize the interference of the ongoing exposure of microorganisms to the adsorbed QACs.

Nitrification and denitrification rates were measured based on the protocols reported by Han et al. (2016). Briefly, 25 mg/L ammonium (NH_4_^+^-N) was added into the flasks to initiate the nitrification tests. DO concentrations were maintained at ~ 5 mg/L. The nitrate nitrogen (NO_3_^−^-N) concentrations in the bulk solutions were periodically determined. The nitrification rates were calculated via the derivation of the NO_3_^−^-N concentrations in the initial 30 min. For the denitrification tests, anoxic conditions were first created by sparging nitrogen gas. NaNO_3_ (and NaAc) were added to make the initial NO_3_^−^-N concentrations at ~ 25 mg/L. Temporal variations of the nitrate concentrations were monitored to determine the denitrification rates. Measurements of NH_4_^+^-N, NO_3_^−^-N and nitrite nitrogen (NO_2_^−^-N) were performed according to the *Standard Methods* (APHA 2012).

Activities of ammonium monooxygenase (AMO), nitrite oxidoreductase (NOR), nitrate reductase (NAR) and nitrite reductase (NIR) were measured after the exposure experiments (Additional file 1: Fig. S1) to evaluate the acute toxicity of QACs to the nitrogen metabolism of activated sludge (Zheng et al. 2012).

### Measurement of microbial intracellular responses

Dehydrogenase activity (DHA) and adenosine triphosphate (ATP) (Han et al. 2016) were monitored to assess the change in heterotrophic metabolism behaviors in response to QAC shock loads. Detailed procedures for DHA and ATP measurements can be found in Additional file 1: Section S2. The generation of reactive oxygen species (ROS), including hydrogen peroxide (H_2_O_2_), superoxide anion (O_2_^⋅−^) and hydroxyl radicals (⋅OH), was detected using ROS detection kits containing H_2_DCF-DA (Life Technology, US) (Additional file 1: Fig. S1). Briefly, fresh sludge samples collected from the MBR were subject to the washing procedures as shown in Additional file 1: Fig. S1. After that, H_2_DCF-DA was added into the sludge samples followed by 20-min incubation at 37 °C in dark. The residual probes were removed by centrifugation and washing (with PBS) thrice. Different kinds of QACs were then added to initiate the exposure experiments (2 h). Oxidation of H_2_DCF by intracellular ROS results in an increase in the fluorescence which was quantified on a multi-mode microplate reader at excitation 488 nm and emission 525 nm (TU-1810, PERSEE, China). Catalase (CAT) and superoxide dismutase (SOD), two key enzymes involved in the antioxidant defense against ROS, were also monitored according to the protocol documented elsewhere (Han et al. 2017).

### Evaluation of membrane fouling

Since the microbial products play an important role in membrane fouling (Malaeb et al. 2013; Meng et al. 2017), consideration was given to the production and release of microbial products in the presence of QACs. Ultrasonication-centrifugation method (Han et al. 2013) was introduced to extract the microbial products following the exposure experiments (Additional file 1: Fig. S1). Proteins and humic acids components, and carbohydrates were determined using the modified Lowry method (Hartree 1972) and phenol–sulfuric acid method (DuBois et al. 1956), respectively. The adhesion and fluidity properties of the microbial products of the activated sludge were evaluated using a quartz crystal microbalance with dissipation (QCM-D) monitoring system (Q-sense E4, Gothenburg, Sweden) (Mei et al. 2014). DI water were initially injected into the chamber for stabilization (frequency drift < 0.2 Hz within 10 min) followed by the introduction of the samples. The frequency (∆*f*) shifts at the third overtone were recorded.

Membrane fouling experiments were conducted in a closed-loop, cross-flow MBR with an effective volume of 1.68 L (Additional file 1: Fig. S1). The MBR tank was divided into a riser zone and two down-comer zones by two baffle plates. The effective membrane filtration area was 240 cm^2^. Predetermined amount (15 μmol/g-SS) of QACs was added into the tank to investigate the acute effects of QACs on membrane fouling. The system was continuously operated for 4 h at a membrane flux of 35 L/(m^2^ h).

## Results

### Influences of QAC shock loads on biodegradation and nitrogen metabolism

**Fig. 1:**
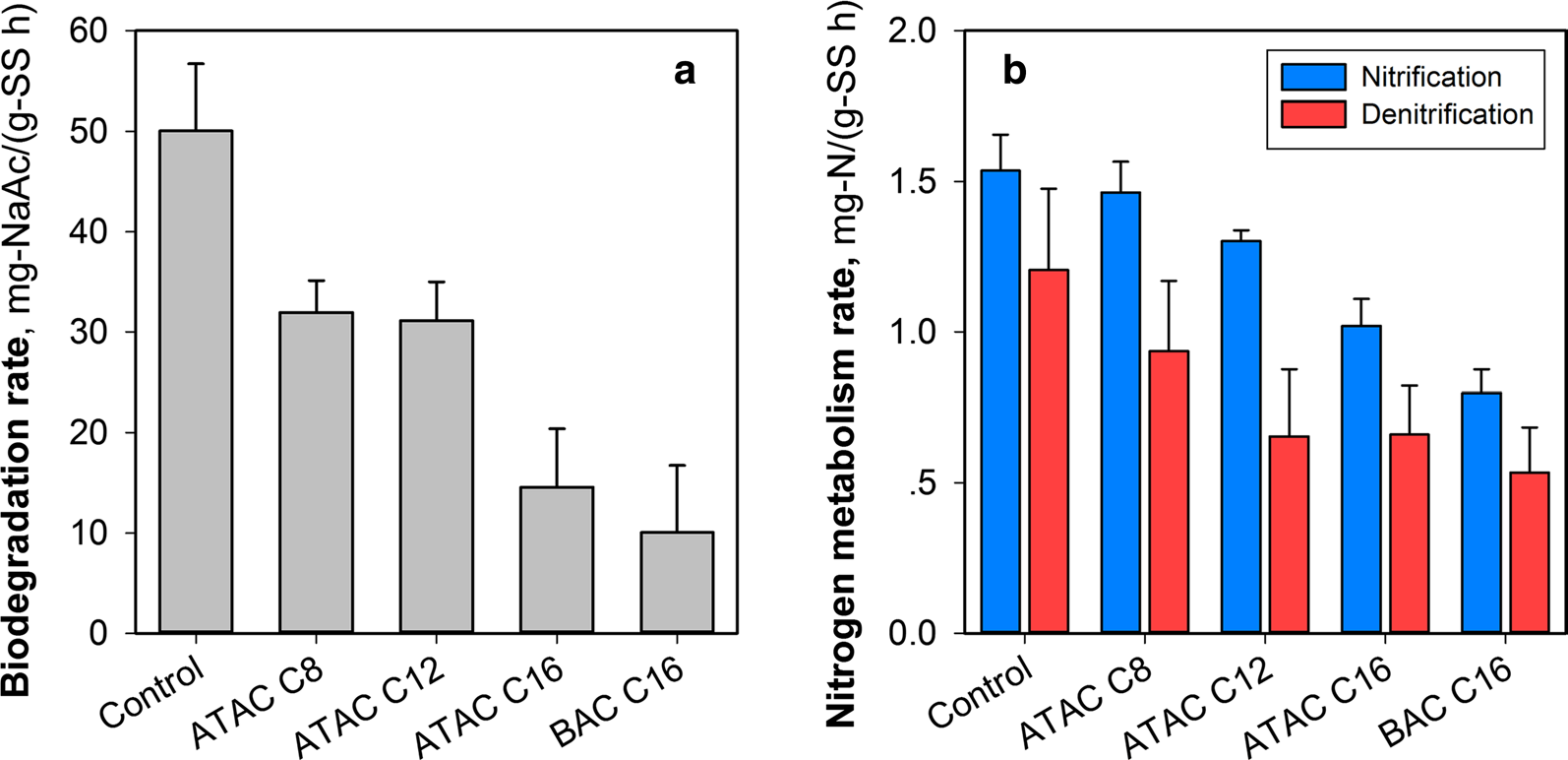
**a** Biodegradation rates and **b** nitrogen metabolism rates of the microorganisms from an MBR following exposure to different kinds of QACs. Experimental conditions: QAC dosage = 0.015 mmol/g-SS and exposure duration = 2 h. Tests were conducted at least in duplicate

Figure 1a summarizes the biodegradation kinetics parameters of organic matters by the microorganisms following exposure to QACs. Compared to the control experiment, it can be observed that QACs significantly affected the microbial activity (*p* < 0.05); i.e., with an increase in the length of alkyl chains, the inhibition became severe, suggesting that a longer alkyl chain could result in higher toxicity to the bacteria. It is also worth noting that the benzalkonium, i.e., BAC C16, demonstrated a higher acute toxicity to the microorganisms than ATAC C16 though they have the identical alkyl chains. This finding is however not surprising because it is alleged that the substitution of a methyl group with a benzyl group can increase the hydrophobicity of QACs (Garcia et al. 2001; Xiao et al. 2008), causing a stronger interaction of QACs with the microorganisms and likely affecting the cell membrane integrity. In MBRs, the deterioration in the biodegradation of organic matters would lead to (i) inferior effluent quality and (ii) an increase in the membrane fouling propensity (Han et al. 2017; Meng et al. 2009, 2017).

Consideration was also given to the impacts of QAC exposure on the nitrification and denitrification processes with the metabolism rates provided in Fig. 1b. Similar trends can be observed for the nitrogen metabolism, i.e., the presence of QACs inhibited both nitrification and denitrification behaviors whilst an increase in the length of alkyl chains caused severer inhibition. For example, with the alkyl chain of the monoalkoniums evolving from C8 to C16, the nitrification and denitrification rates of the samples decreased from (1.46 ± 0.10) and (0.94 ± 0.23) mg-N/(g-SS h) to (1.02 ± 0.09) and (0.66 ± 0.16) mg-N/(g-SS h), respectively. The aggravated toxicity induced by the terminal benzyl group was also observed, indicating that the changes in the nitrogen metabolism due to the transient QAC shock loads are generally in agreement with that of the biodegradation of organic matters.

**Fig. 2:**
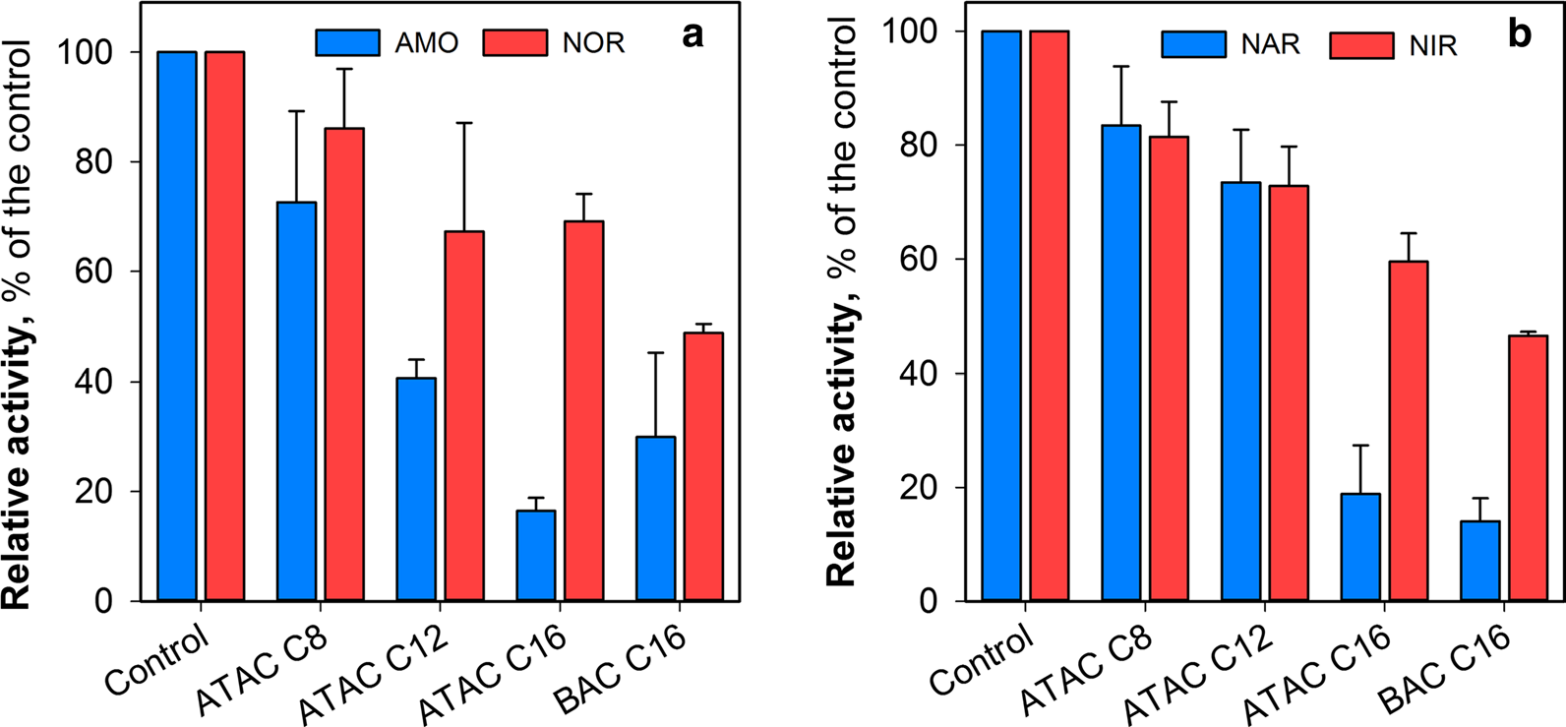
Variations of **a** AMO and NOR, and **b** NAR and NIR activities of the sludge samples following 2-h exposure to QACs. Tests were conducted at least in duplicate

Further analyses of the activities of key enzymes, including AMO and NOR involved in nitrification process and NAR and NIR in denitrification process, were performed because these key enzymes played a vital role in the biological nitrogen metabolism (Zheng et al. 2012). As can be seen from Fig. 2, the activities of AMO, NOR, NAR and NIR were inhibited following 2-h exposure to QACs, which largely accounted for the changes of the kinetics of nitrification and denitrification (Fig. 1b). The penetration of alkyl chains into the cell membrane might cause damage to these enzymes since they are bound to the inner cytoplasmic surface of the bacterial membrane (Han et al. 2016; Krause and Nealson 1997).

### Microbial intracellular responses to the transient shock loads

**Fig. 3:**
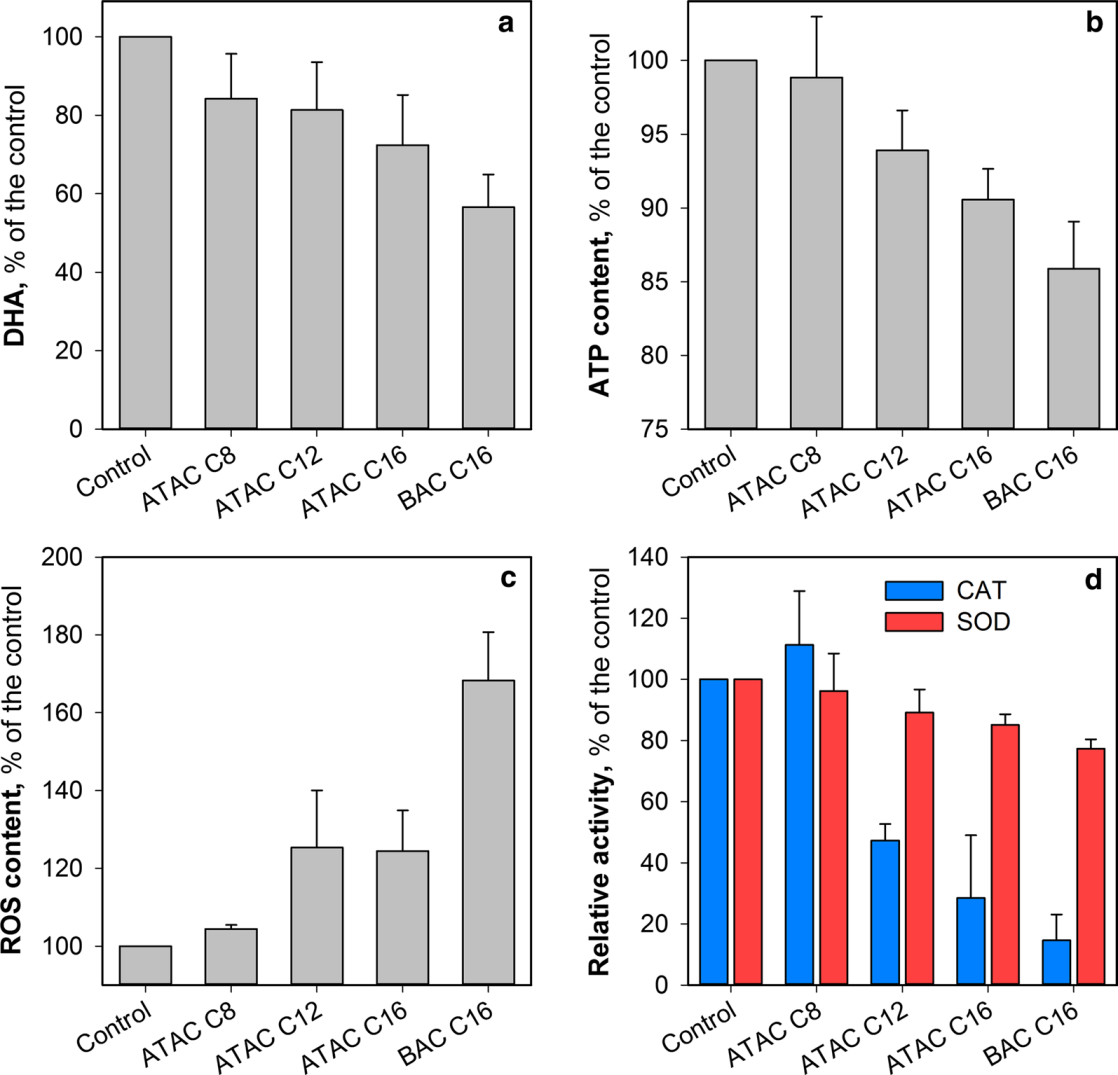
Variations of **a** DHA, **b** ATP content, **c** ROS content and **d** CAT and SOD activities of the sludge samples following 2-h exposure to QACs. Tests were conducted at least in duplicate

DHA and ATP were chosen as comprehensive indicators to evaluate the microbial response to the environmental stress induced by QACs. DHA is involved in the tricarboxylic acid (TCA) cycle while ATP is generated during oxidative phosphorylation. As shown in Fig. 3a, compared to the control test, DHA of the sludge was downregulated on exposure to QACs and further decreased with an increase in the alkyl chain length. The inhibition of DHA is consistent with the changes of specific oxygen uptake rate (SOUR) (Additional file 1: Fig. S2), confirming that QACs affected the microbial aerobic respiratory and consequently the TCA cycle.

The variation of ATP content demonstrated a similar changing pattern to that of DHA (Fig. 3b). ATP production was obviously inhibited after exposure to ATAC C16 (90.6% of the control) and BAC C16 (85.9% of the control) compared to ATAC C8 (98.8% of the control, *p* > 0.05). The decrease in ATP production might be associated with the disruption of microbial cells by QACs, which consequently caused damage to the enzymes responsible for energy production [e.g., adenylate kinase (ADK)] since ATP is generated from the adenosine diphosphate (ADP) via catalysis of ADK during oxidative phosphorylation. Moreover, the substitution of the methyl group with a benzyl group also led to a more significant inhibition on ATP production, further confirming that derivatization of QACs (e.g., grafting of benzyl groups) could aggravate the adverse effects of QACs on microorganisms.

During oxidative phosphorylation process, ROS are generated from sequential univalent reduction (and activation) of oxygen (Eqs. 1–3) (Scandalios 2002). It can be observed from Fig. 3c that there is no significant difference (*p* > 0.05) in ROS response between the control test and ATAC C8, while ROS content in the sludge sample following 2-h exposure to BAC C16 is ~ 1.7 times that of the control. The presence of QACs (e.g., ATAC C12 and ATAC C16) could cause oxidative stress in microbial cells in addition to the physical damage to cell membrane by the alkyl penetration. It is also worth noting that BAC C16 induced higher ROS production than ATAC C16, suggesting that the benzyl group could exert additional intracellular stress. The elevated production of ROS could partially explain why BAC C16 caused more negative influences on the microbial viability compared to ATAC C16 though they have the same alkyl chain length.

1 $$ {\text{O}}_{ 2} \mathop{\longrightarrow}\limits^{{e^{ - } }}{\text{O}}_{ 2}^{ \cdot - } $$

2 $$ {\text{O}}_{2}^{ \cdot - } {\text{ + O}}_{2}^{ \cdot - } \mathop{\longrightarrow}\limits_{\text{SOD}}^{{ 2 {\text{H}}^{ + } }}{\text{H}}_{ 2} {\text{O}}_{ 2} + {\text{O}}_{2} $$

3 $$ {\text{Fe}}^{\text{II}} + {\text{H}}_{ 2} {\text{O}}_{ 2} \to {\text{Fe}}^{\text{III}} {\text{ + OH}}^{ - } + {\text{HO}}^{ \cdot } $$

Microbial cells are equipped with vital enzymes (e.g., CAT and SOD) in the antioxidant defense against ROS (Han et al. 2016; Scandalios 2002). Changes in the activities of CAT and SOD in the presence of various QACs are shown in Fig. 3d. It can be observed that, in comparison with the control test, there is no significant deterioration for both CAT and SOD activities when the sludge sample was exposed to ATAC C8. In contrast, with an increase in alkyl chain length from C8 to C16, the inhibition on both CAT and SOD activities became obvious. This might consequently induce an imbalance between the generation of reactive oxygen species and a biological system’s ability to readily detoxify the reactive intermediates and/or to repair the resultant damage (Nakata et al. 2011). The accumulation of ROS triggered by QACs would cause damage to the cellular components and even result in cell apoptosis (and death) (Ceragioli et al. 2010).

### Microbial product release and fouling propensity

**Fig. 4:**
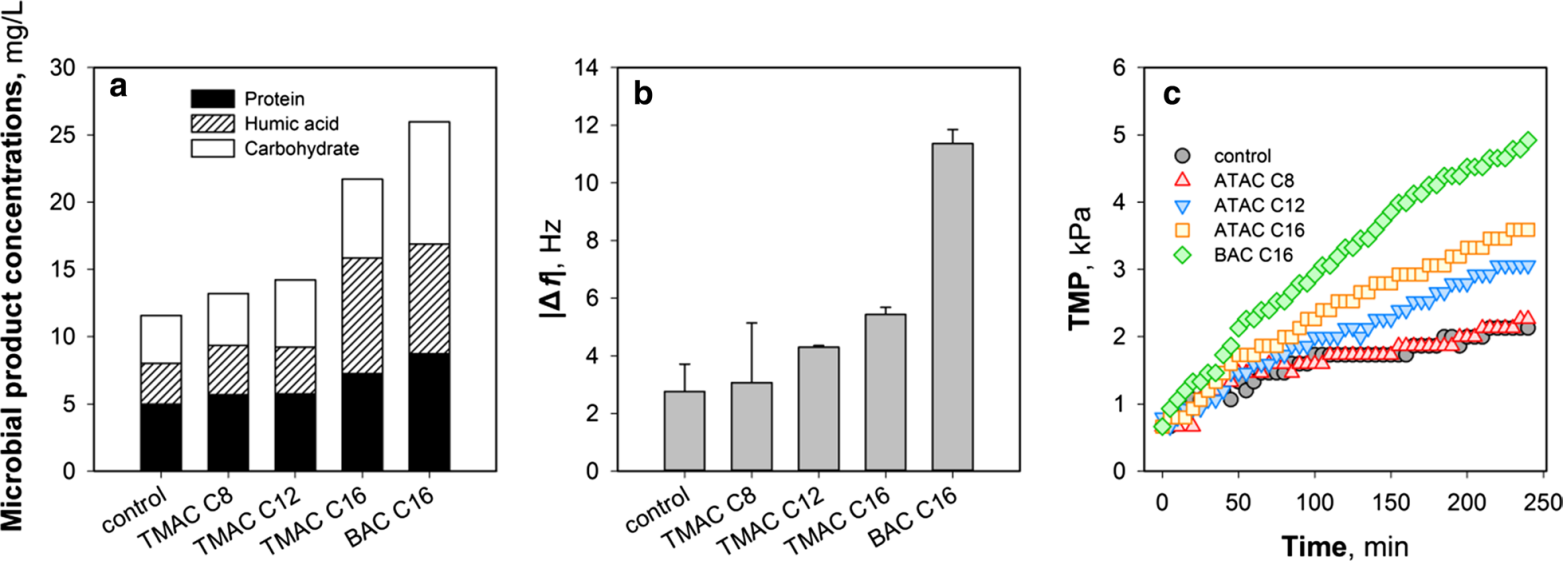
Variations of **a** microbial products and **b** adhesion and fluidity properties of the microbial products following exposure of the samples to different kinds of QACs for 2 h. The adhesion and fluidity properties are evaluated by the frequency shifts (∆*f*) at the third overtone after the adsorption of microbial products on QCM-D sensors. **c** Time-course results of the transmembrane pressure (TMP) in an MBR under the shock loads of different kinds of QACs

Release of microbial products in the presence of QACs was also examined in this study, since the accumulation of microbial products can not only deteriorate the effluent quality but also result in severe membrane fouling (Meng et al. 2017; Wang et al. 2009). In Fig. 4a, it can be observed that the total amounts of microbial products slightly increase with the microorganisms exposed to ATAC C8 and ATAC C12 while those from sludge samples in the presence of ATAC C16 and BAC C16 are significantly higher than the control. This finding indicates that an increase in the alkyl chain length might (i) facilitate the dissociation of extracellular microbial products from the cell walls and (ii) aggravate the damage to cell integrity leading to an increased release of the intracellular organic matters. Moreover, the total amount of microbial products of the sample following exposure to BAC C16 was higher than ATAC C16. This suggests that the stronger interaction of BAC C16 with microorganisms and the elevated production of ROS (Fig. 3c) likely caused severer damage to cell membrane (and walls), resulting in the increased release of organic matters.

To further elucidate the acute effects of QACs on membrane fouling, the adhesion properties of the microbial products were evaluated using QCM-D. The frequency shifts (∆*f*) after the introduction of microbial products for 20 min are shown in Fig. 4b (the evolution of ∆*f* could be found in Additional file 1: Fig. S3). It is evident that microbial products extracted from the biomass exposed to longer alkyl-chain QACs have higher adsorption propensity as indicated by the higher frequency shifts (∆*f*) (Fig. 4b). Especially, ∆*f* of the microbial products for BAC C16 was the largest, suggesting the highest fouling propensity.

A lab-scale cross-flow filtration reactor was then used to evaluate the membrane fouling propensity of an MBR under the shock loads of different QACs. Figure 4c shows that TMP gradually increases as a function of filtration time. Notably, the TMP increase rates follow the order of BAC C16 > ATAC C16 > ATAC C12 > ATAC C8 ≈ control, which is in agreement with the results in Fig. 4a and b, confirming that the strong release of microbial products would cause a higher membrane fouling propensity.

## Discussion

**Fig. 5:**
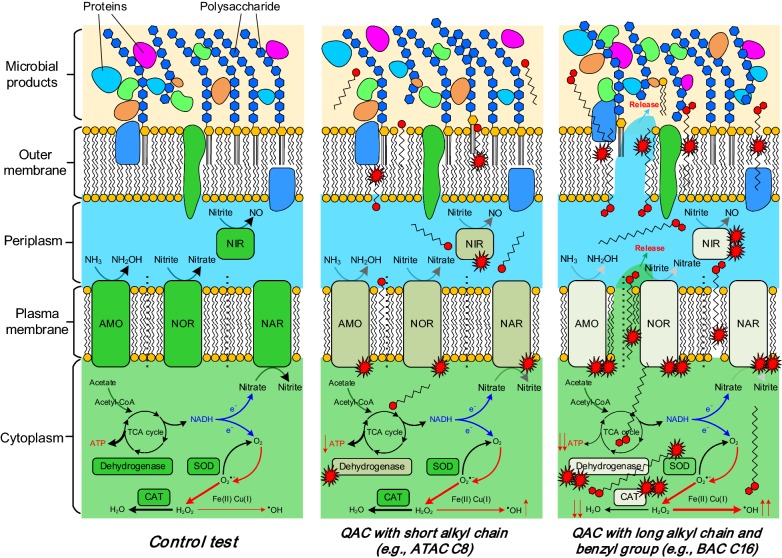
A schematic representation of the acute toxicity induced by the transient shock loads of different kinds of QACs. The dash lines in plasma membranes indicate that AMO, NOR, NAR and NIR might be assigned to different phylotypes. The gradually lightened color of AMO, NOR, NAR, NIR, dehydrogenase, and CAT in the three panels indicates an increased negative influence. The line thickness of the arrows associated with ATP and H_2_O_2_ (transformed to either H_2_O or ⋅OH) is proportional to their quantity

Results in this study clearly demonstrate that the transient shock loads of QACs would result in adverse impacts on the activity of the MBR sludge and, consequently, deteriorate the process performance. With regard to the understanding on the interaction of QACs with the microorganisms (Chen et al. 2014; Christen et al. 2017; Di Nica et al. 2017), a schematic representation describing the acute toxicity of different kinds of QACs is given in Fig. 5.

It has been accepted that the interaction of QACs with microorganisms commences with two steps, i.e., adsorption, and alkyl chain penetration (Ismail et al. 2010; Lambert and Pearson 2000; Zhang et al. 2015). This should be ascribed to the permanently charged polyatomic ions of the structure NR_4_^+^ giving QACs the property to be readily adsorbed on the cell walls (and membranes) that are normally negatively charged (Zhang et al. 2016, 2017). Additional file 1: Fig. S4a shows the change of zeta potentials of the sludge following exposure to different kinds of QACs. It can be observed that a longer alkyl chain and benzyl groups could increase the hydrophobic interaction of QACs with the cell membranes (i.e., phospholipid bilayers), resulting in the zeta potential of the sludge flocs being less negative and the surface more hydrophobic (Additional file 1: Fig. S4b). The enhanced interaction could facilitate the subsequent physical penetration. Meanwhile, the adsorbed QACs are expected to lower the surface tension (and interfacial tension) between the loosely-bound microbial products and the bulk solution (Fig. 5). As shown in Fig. 4a, the release of extracellular proteins and polysaccharides has been enhanced with the alkyl chain increased from C8 to C16, which is largely in agreement with the aggravated membrane fouling (Fig. 4c). It should be noticed that exposure to BAC C16 might result in (i) severe damage of cell integrity and (ii) leakage of intracellular organic matters as the adhesion properties of the “microbial products” changed substantially (Fig. 4b). As such, the fouling propensity of the sample after exposure to BAC C16 was the highest (Fig. 4c).

The hydrophobic alkyl chains of the adsorbed QACs would penetrate the phospholipid layers (Wessels and Ingmer 2013), induce excessive oxidative stress (Nakata et al. 2011), cause damage to key enzymes (e.g., AMO and SOD), and even lead to cell apoptosis (and death) (Xiao et al. 2008). It has been reported that the penetration of QACs might be associated with the loss of cell membrane osmoregulation and the dissipation of proton motive force (Tezel and Pavlostathis 2015), and the profound consequences include the change in ATP synthesis (Fig. 3b) (Rao et al. 2008). While QACs demonstrate a similar trend of acute toxicity (i.e., a longer alkyl chain and/or benzyl group indicating higher fungistatic/bacteriostatic capabilities) to (i) the key enzymes on plasma membranes involving nitrification and denitrification and (ii) the dehydrogenase in cytoplasm coordinating TCA cycle (and biodegradation), different mechanisms might be involved in the generation of the excessive oxidative stress for ATAC C8 and ATAC C16 (and BAC C16). Results in Fig. 3c and d indicate that ATAC C8 can not inactivate the key enzymes in the antioxidant defense against ROS (Eqs. 2 and 4), thus leading to a low ROS content following 2-h exposure (Fig. 3c). In contrast, the introduction of QACs with longer alkyl chains and/or benzyl groups (e.g., BAC C16) caused severe inhibition on CAT though SOD was less affected, causing the accumulation of intermediate oxidants (such as H_2_O_2_) in the cytoplasm (Fig. 3c). These oxidants and the free radicals (⋅OH) activated by Fe(II)- and/or Cu(I)-rich cytochromes (Eq. 3) were capable of destroying intracellular structures. The cell lysis would increase the concentrations of “microbial products”, affect the effluent quality and membrane filtration and, finally, deteriorate the process performance.

4 $$ 2 {\text{H}}_{ 2} {\text{O}}_{ 2} \mathop{\longrightarrow}\limits^{\text{CAT}}{\text{H}}_{ 2} {\text{O + O}}_{ 2} $$

The increasing utilization of QACs inevitably leads to their environmental release into wastewater treatment plants (and MBRs). This study clearly shows that the transient shock loads of QACs in the wastewater can cause acute toxic effects on microbial metabolism, resulting in the inhibition of biodegradation and nitrogen metabolisms. The severe consequences might involve (i) the elevated concentrations of chemical oxygen demand (COD) and nitrogen in the effluent and (ii) sharp increase in TMP and more frequent membrane cleaning. Therefore, special attention should be paid to the performance of biological wastewater treatment processes subject to the shock loads of industrial streams while the acclimatization and versatility of MBRs treating the frequent shock loads in long-term operation need further investigation.

This study also demonstrates that different kinds of QACs have different acute toxicity to the microorganisms in MBRs. Specifically, QACs with longer alkyl chains and/or benzyl groups bonded to the nitrogen atom can induce a more severe damage to cell integrity and microbial viability. Identification of QAC species in the influent wastewater could provide useful information predicting their potential influences on the process performance though further studies on the synergistic effects of environmental factors (e.g., pH and temperature) on the toxicity are still needed. Attention should be also paid to the release of organic matters from microorganisms under the transient shock loads of QACs. The released organic matter together with the inhibited biodegradation of contaminants not only increases the effluent COD but also potentially leads to the production of disinfection byproducts (during effluent disinfection) in the biological wastewater treatment processes.

## Additional file

**Additional file 1.** Text sections (Section S1–S3), and Figures S1–S3 are included.
