# Supplementary material for: Microbial responses to transient shock loads of quaternary ammonium compounds with different length of alkyl chain in a membrane bioreactor
Source: AMB Express. 2018 Jul 17;8:118. doi: 10.1186/s13568-018-0649-5 (PMC6049845; doi:10.1186/s13568-018-0649-5)
Supplement: Supplementary file 1 — Additional file 1. Text sections (Section S1–S3), and Figures S1–S3 are included. [file 13568_2018_649_MOESM1_ESM.docx]

**AMB Express**

**Additional file 1**

**Microbial responses to transient shock loads of quaternary ammonium compounds with different length of alkyl chain in a membrane bioreactor**

Xingran Zhang^1§^, Jinxing Ma^2§^, Mei Chen^1^, Zhichao Wu^1^, Zhiwei Wang^1, *^

^1^State Key Laboratory of Pollution Control and Resource Reuse, Shanghai Institute of Pollution Control and Ecological Safety, School of Environmental Science and Engineering, Tongji University, 1239 Siping Road, Shanghai 200092, China

^2^UNSW Water Research Centre, School of Civil and Environmental Engineering, University of New South Wales, Sydney, NSW 2052, Australia

*Corresponding author. Tel/Fax: +86(21)65980400; E-mail address: zwwang@tongji.edu.cn (Z.W. Wang) § These two authors contribute equally to this work

**Contents**

**Text Sections**

**Section S1.** Information about the pilot-scale MBR where the sludge inoculums were collected

**Section S2.** Analytical methods of DHA activity and ATP content.

**Section S3.** Analytical methods of SOUR.

**Figures**

**Fig. S1.** Schematic representation of the experimental procedure.

**Fig. S2.** Variations of SOUR of sludge samples following 2-h exposure to different QACs.

**Fig. S3.** Frequency and dissipation shifts in determining the adsorption of microbial products by QCM-D.

**Fig. S4.** Variation of (a) zeta potentials and (b) contact angles of sludge samples after 2-h exposure to QACs.

**Section S1.** Information about the pilot-scale MBR where the sludge samples were collected

The sludge samples were collected from a pilot-scale anoxic/oxic membrane bioreactor (A/O – MBR) fed with municipal wastewater. The reactor has an oxic zone with effective volume of 30 L and an anoxic zone with effective volume of 22 L. Six flat-sheet membranes (SHZZ-MF, Zizheng Environment Inc., Shanghai, China) were installed in the oxic zone with total effective filtration area of 0.63 m^2^. The mean pore size of the membrane is 0.2 μm. Membrane flux is maintained at 15 (L/m^2^·h). The hydraulic time (HRT) is maintained at 6.6 h and sludge retention time (SRT) is 60 d through daily discharge of excess sludge. The concentration of activated sludge in MBR is maintained at about 10 g/L.

**Section S2.** Analytical methods of DHA activity and ATP content.

In brief, Dehydrogenase (DHA) activity was assessed by 2-(piodophenyl)-3-(p-nitrophenyl)-5-phenyl tetrazolium chloride (INT)-DHA method (Han et al., 2016) 1 mL of INT solution (2 g/L) was added into 0.5 mL microorganism samples after washing (See Scheme 1). After incubation in the dark (37 ^o^C) for 30 min, ethyl acetate was used to extract the insoluble red triphenyl formazan (TF) crystal. The supernatant was obtained by centrifugation (8000*g*, 5 min) and measured at 490 nm on a spectrophotometer (TU-1810, PERSEE, China).

The content of adenosine triphosphate (ATP) was determined using the assay kit (Promega, America). Sludge samples were centrifuged (8000*g*, 5 min) and washed twice. In order to maintain the concentration of cells in a proper range for luminescence testing, the pellets were resuspended and diluted for 20 times using DI water. 100 μL of prepared detection reagent was added into 100 μL sludge sample. After mixing for 2 min to induce cell lysis and incubation for 10 min to stabilize the luminescent intensity, the luminescent intensity of each sample was measured by a multi-mode microplate reader (TU-1810, PERSEE, China).

**Section S3.** Analytical methods of SOUR.

Specific oxygen uptake rate (SOUR) of this resuspended samples was measured by respirometry according to Huang et al., (2015). Synthetic wastewater (including 100 mg/L NaHCO_3_, 120 mg/L CH_3_COONa, 76 mg/L NH_4_Cl and 99 mg/L NaNO_2_) was used as the nutrient media and dissolved oxygen (DO) was monitored by DO meter (HQ40d, Hach, America). The slope of DO versus time curve normalizing by volatile suspended solids (VSS) concentration is considered as the SOUR of each sample.


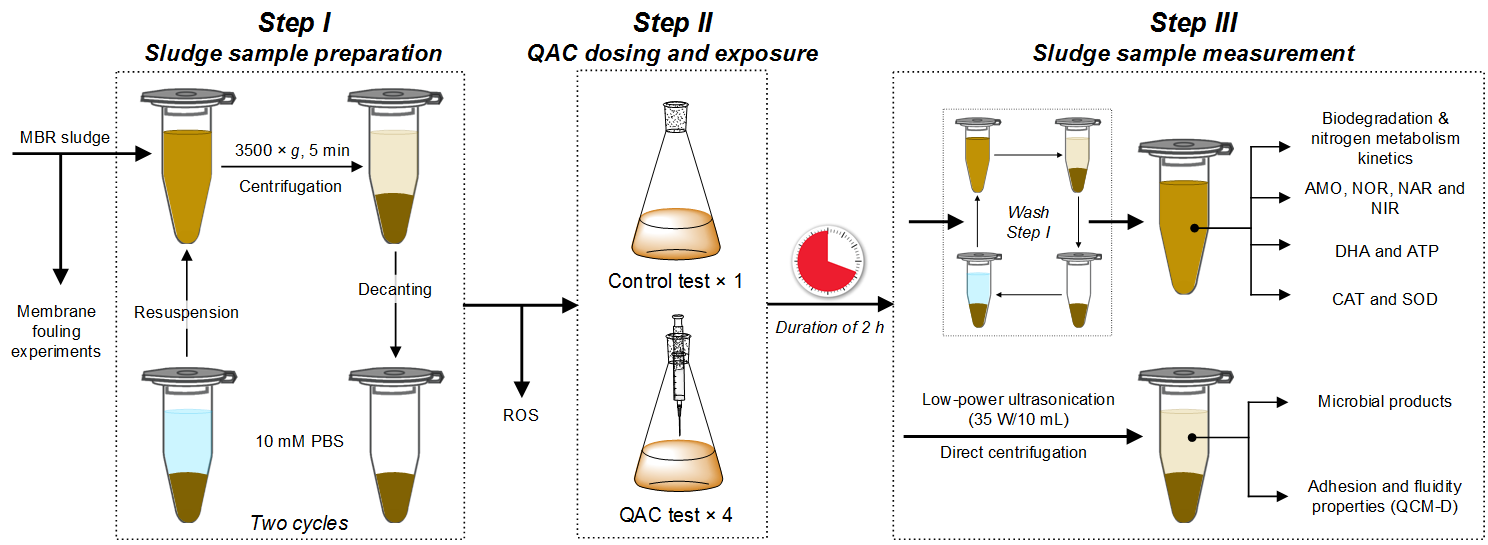


**Fig. S1.** Schematic representation of the experimental procedure.

**Fig. S2.** Variations of SOUR of sludge samples following 2-h exposure to different QACs.

**Fig. S3.** Frequency and dissipation shifts in determination of the adsorption propensity of microbial products using QCM-D.


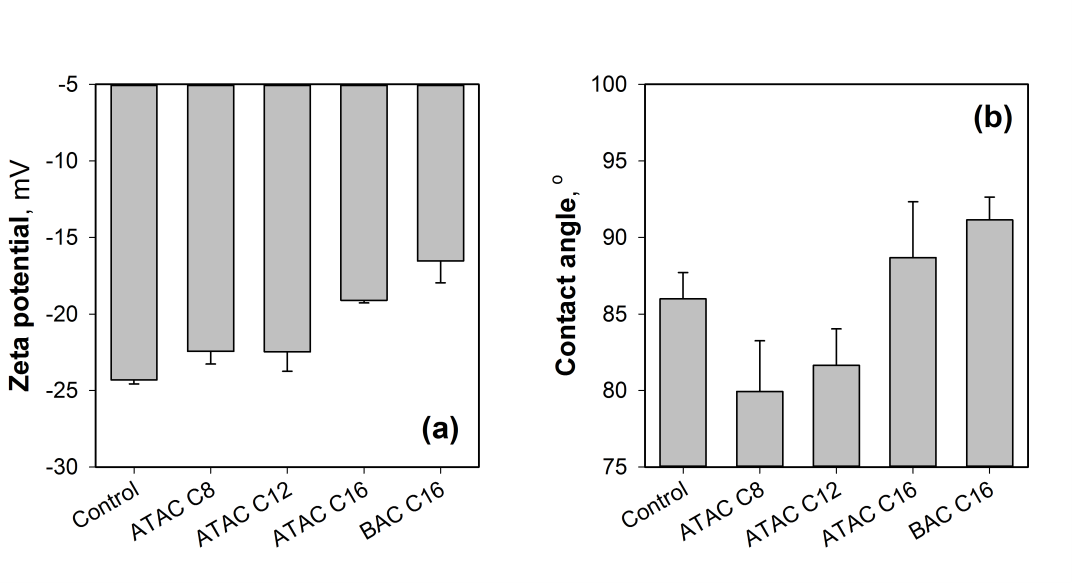


**Fig. S4.** Variation of (a) zeta potentials and (b) contact angles of sludge samples after 2-h exposure to QACs. Zeta potentials and contact angles of sludge samples were measured according to standard procedures according to Lei et al., (2016).

**References**

Han, X., Wang, Z., Wang, X., Zheng, X., Ma, J., Wu, Z., 2016. Microbial responses to membrane cleaning using sodium hypochlorite in membrane bioreactors: Cell integrity, key enzymes and intracellular reactive oxygen species. Water Res. 88, 293-300.

Huang, J., Wang, Z., Zhang, J., Zhang, X., Ma, J., Wu, Z., 2015. A novel composite conductive microfiltration membrane and its anti-fouling performance with an external electric field in membrane bioreactors. Sci. Rep.-UK, 5, 9268.

Lei, Q., Li, F., Shen, L., Yang, L., Liao, B.,Lin, H., 2016. Tuning anti-adhesion ability of membrane for a membrane bioreactor by thermodynamic analysis. Bioresour. Technol. 216, 691-698.
